# 小细胞肺癌的诊疗进展

**DOI:** 10.3779/j.issn.1009-3419.2011.10.09

**Published:** 2011-10-20

**Authors:** 少华 周, 志龙 赵

**Affiliations:** 116001 大连，大连大学附属中山医院胸外科 Department of Thoracic Surgery, Zhongshan Hospital, Affiliated to Dalian University, 116001 Dalian, China

小细胞肺癌（small cell lung cancer, SCLC）是肺癌中恶性程度最高的一种类型，具有进展快、转移早、易复发等特点，约占新发肺癌15%-20%，其发生与长期吸烟有密切关系；SCLC未经治疗者的中位生存期（median survival time, MST）仅为2个月-4个月^[1]^；治疗后，局限期患者的MST约为15个月-20个月，广泛期患者的MST为8个月-13个月^[2]^。同其它类型肺癌相比，SCLC对化疗和放疗比较敏感，然而，由于存在高复发率和耐药率，因此在治疗方面仍然面临诸多挑战。

近年，针对SCLC的研究不断深入，有关新诊疗技术和策略的临床结果不断涌现。本综述旨在对此进行系统回顾。

## 1 SCLC的诊断和分期

2009年，国际抗癌联盟（Union for International Cancer Control, UICC）建议，SCLC与非小细胞肺癌均采用TNM分期^[3]^。根据这一分期系统，可以筛选出早期且适宜手术的患者。

美国退伍军人医院肺癌研究小组^[4]^制定的SCLC分期系统使用久远，且简单易行。根据此分期，如果肿瘤局限于一侧胸腔且能被纳入一个放射治疗野即为局限期（limited disease, LD），如果肿瘤超出局限期的范围即为广泛期（extensive disease, ED），其中前者约占30%-40%。

美国国立综合癌症网络指南2012年第2版建议^[5]^：SCLC分期前的初始评估包括：病史和体格检查；病理学复习；全血细胞计数、电解质（包括血钙）、肝肾功能等生化检测；胸、肝脏和肾上腺CT，如果必要应进行增强扫描；头部MRI（优先选择）或CT检查；如果考虑是局限期病变，应行PET/CT扫描，对于发现的病灶，推荐进行病理学确认，以明确分期，如无法进行PET/CT扫描时应进行骨扫描；戒烟辅导和干预。指南将SCLC的两种分期融合在一起对其诊断和治疗进行阐述。

对于LD伴有胸水者，如果胸穿无法明确性质，应考虑使用胸腔镜。大多数胸水是由于肺癌转移所致，但如果胸水较少而不能通过影像导引取样，则胸水不应被纳入分期。如果细胞学检查阴性，且不是血性或渗出性，则临床可以判定胸水与癌症并不直接相关，且不应成为ED的证据。

如果PET/CT或骨扫描显示有异常摄取，应行骨放射学检测；对于仍不能确定的，可考虑行MRI检测。

临床分期为T1-2N0的LD患者，可行PET/CT扫描，以确认远处或纵隔转移，之后应进行病理学纵隔分期，手段包括纵隔镜检查术、纵隔切开术、支气管或食管内镜下超声引导活检、电视胸腔镜。如果内镜下淋巴结活检结果为阳性，则不需要采取其它分期措施。

如果外周血涂片显示有核红细胞、嗜中性白细胞或血小板减少，则应行单侧骨髓穿刺活检。

SCLC的预后与患者治疗前的临床特征直接相关。具有良好体能状态（performance status, PS）、女性、局限期患者预后较好^[6-9]^；研究^[7, 10]^发现血清钠浓度、碱性磷酸酶、乳酸脱氢酶等生化因子是独立的预后影响因子。此外，化疗后肿瘤缓解率高和患者耐受性好均提示预后良好^[5]^。

## 2 治疗

SCLC治疗的演变历经以手术为主，放疗替代手术、以化疗为主，再到接受手术作为早期治疗的组成部分几个阶段。不可否认，化疗仍然是SCLC治疗的核心。

### 2.1 一线治疗

#### 2.1.1 化疗

目前，SCLC一线化疗主要是以铂类药物为基础的联合治疗，最常用的是依托泊苷+顺铂或卡铂（EP或CE方案）。

NCCN指南建议^[5]^：无论是LD还是ED，均应接受4个-6个疗程的顺铂/卡铂+依托泊苷/伊立替康联合治疗；对于部分或完全缓解的SCLC患者，不推荐维持疗法。对于年龄大、心肾功能差且不能耐受顺铂的患者，CE方案能够产生相似的疗效且毒性较小。而依托泊苷口服的疗效略逊于静脉给药，并且毒性增加^[11]^。

近期一项针对908例ED期患者的Ⅲ期随机研究^[12]^显示，培美曲赛联合卡铂的疗效不如CE方案，中位总生存期分别为8.1个月和10.6个月，中位无进展生存期分别为3.8个月和5.4个月。3级-4级白细胞减少的发生率较CE方案低，而贫血的发生率略高，血小板减少症发生率两者相似。

对比吉西他滨和卡铂（GC）方案同EP方案的研究^[13]^显示，在生存率、反应率和进展时间方面，两种方案无明显差异，而毒副作用却有不同表现。因此，对比GC和CE方案的研究似乎更有实际意义。不过，GC方案对于含有非小细胞肺癌成分的SCLC可能更为适合。

日本临床肿瘤学会的研究显示，伊立替康和顺铂对比EP方案治疗ED患者，前者可明显延长生存时间。而在美国患者中却没有得出相同结果^[14]^，提示民族之间存在基因变异。因此，我国应该广泛开展临床相关研究，制定适合我国国民的治疗方案。

#### 2.1.2 放疗

SCLC对放疗比较敏感。理论上讲，放射剂量越高，对肿瘤杀伤力越强，但放射损伤发生率及程度也随之增高。

一项荟萃分析^[15]^结果虽然未能得出每天1次常规分割放疗的最佳总剂量，但基于现代放疗技术，给予总剂量达70 Gy是安全的。从肿瘤生物学行为方面看，胸腔照射的剂量最少应为40 Gy-50 Gy；40 Gy以下不能保证肿瘤得到控制；放疗60 Gy以上，多采用超分割，但其对肿瘤控制率及放射毒副作用有待进一步研究，在临床实施中应依据个体差异权衡利弊。Takada等^[16]^实施的一项Ⅲ期临床研究显示：基于EP化疗，同步放疗组的治疗效果要明显优于序贯放化疗组，但同步治疗的毒副作用更大。De Ruysscher^[17]^指出，从长期生存率角度出发，疗程 < 30 d的放疗适用于与所有放化疗结合的治疗方案。

如果LD患者身体状况允许，放疗应与化疗联合应用。并且，首选三维适形放射技术，经选择的患者可采用调强放疗，而四维成像和/或其它可行技术也可用以评估肿瘤的活动，运动管理的目标是控制活动 < 1 cm或适当地增加计划靶区边缘^[5]^。

#### 2.1.3 手术治疗

最初的研究^[18]^显示：LD-SCLC患者手术后生存率较低，因此手术治疗被放疗取代。然而，放化疗后，SCLC局部复发率仍然很高；理论上，手术可根治没有转移的局限性病灶，也是最有效的治疗反应评估工具，同时可以避免由于细针穿刺误诊类癌和含有非小细胞成分的混合癌，所以手术仍然受到部分医师的青睐。

国内外回顾性研究^[19-32]^显示，手术介入Ⅰ期甚至是Ⅱ期和Ⅲ期SCLC的综合治疗后，取得了更好的治疗效果。其中，Schreiber等^[32]^的研究结果最值得重视，不仅因为作者是放射肿瘤学医师，还因为其纳入的样本量非常大。该研究分析了SEER注册的14, 179例LD-SCLC患者，分为局限性（T1-2Nx-0）和区域性（T3-4Nx-0）两类。手术患者863例，不论局限性还是区域性，都从手术治疗中明显获益，MST分别为42个月、22个月，非手术者则分别为15个月、12个月。针对N0、N1和N2病变，手术者MST分别为42个月、29个月、19个月，非手术者分别为15个月、14个月、12个月。术后放疗不能使N0、N1患者获益，但能延长N2患者生存。多因素分析显示，包含肺叶切除术的综合治疗可以给LD患者带来最大的生存益处。

迄今唯一一项多中心随机对照研究^[33]^评估了328例LD患者，发现手术不能提高总生存率。但是该研究存在不足：没有纳入外周结节病灶；T1-2N0M0患者仅13例；统计功效较低。

根据NCCN指南^[5]^，SCLC患者中，Ⅰ期不足5%。对于T1-2N0M0患者，推荐行肺叶切除和纵隔淋巴结清扫或采样术。术后淋巴结无转移者，单纯化疗；淋巴结转移者，则推荐同步化疗和纵隔放疗。

### 2.2 二线治疗

一线治疗后，根据反应可将患者分为3类：①敏感型：缓解>90天；②耐药型：90天内复发；③难治型：无缓解。LD-SCLC一线治疗的反应率为70%-90%，ED-SCLC的反应率为50%-60%；初始治疗1年内，约80%的LD和几乎全部的ED患者复发或进展^[34]^。由此可见，二线治疗效果关乎SCLC患者的最终生存情况。

拓扑替康联合卡铂治疗复发SCLC的Ⅰ期临床研究显示^[35]^，部分反应率为17.2%，MST为11.3个月，1年生存率为50%。一项随机研究^[36]^发现：同单纯支持治疗复发SCLC相比，支持治疗同时口服拓扑替康者生存明显延长（MST：25.9周*vs* 13.9周）。两项研究均显示，二线化疗患者的耐受性好，生存质量高。另外一项研究中^[37]^，对联合铂类一线化疗敏感的SCLC患者，随机接受氨柔比星和拓扑替康单药维持治疗，MST为9.2个月和7.6个月，氨柔比星还显示出较低的血液毒性。

Chen等报告^[38]^：伊立替康联合卡铂治疗复发SCLC，反应率为50%，MST为10个月，显示了一定的疗效。

初治治疗后2个-3个月内复发且PS评分为0分-2分者，可以选择异环磷酰胺、紫杉醇、多西紫杉醇、吉西他滨、伊立替康、拓扑替康；而2个-3个月至6个月内复发者，可选用拓扑替康口服或静注、紫杉醇、多西紫杉醇、吉西他滨、伊立替康、长春瑞滨、口服依托泊苷、环磷酰胺/多柔比星/长春新碱（CAV）；而超过6个月复发者，应用初始药物。对于PS评分较差者，应考虑减少剂量、应用生长因子^[5]^。

### 2.3 老年患者的治疗

所有新诊断的SCLC患者中，75%患者年龄≥60岁，20%患者年龄≥75岁^[39]^。许多老年患者没有接受标准治疗，其预后比年轻并接受治疗的患者差。

老年患者不接受化疗的原因有：本人或家属拒绝治疗﹑较短的预期寿命﹑高龄﹑身体状态差和伴发疾病^[40, 41]^。尽管PS评分较好，老年患者（≥75岁）接受环磷酰胺+阿霉素+依托泊苷或EP方案化疗，约2/3患者因发生严重毒性反应而不能完成系统化疗^[41]^。因此，对于老年患者，除了需要完善指标、细化化疗指证外，还要开发低毒药物以及合理的化疗策略。

对于肾功衰竭的老年患者，是否可以化疗以及如何化疗？有学者对该问题进行了探索。1例老年ED-SCLC患者伴有尿毒症，血液透析的同时，接受了多种方案的化疗，生存期超过2年^[42]^。有研究结果^[43]^显示，对于伴有肾功衰竭的患者，给予化疗标准剂量的2/3是恰当的。对于此类患者，不能因为血液透析而放弃化疗，但应注意更多毒副作用的发生。化疗的同时监测血药浓度更加安全。

对器官和身体功能状况良好的老年患者，应给予含铂化疗；伴有较差体能状态或其它严重疾病者，可选择培美曲赛、伊立替康、拓扑替康等低毒性药物，同时适当减低剂量是必要的^[5, 41]^。

### 2.4 其它治疗

#### 2.4.1 预防性颅脑照射（prophylactic cranial irradiation, PCI）

SCLC患者中，60%以上将出现可检测到的、有症状的脑转移灶。而PCI能够降低约20%的脑转移发生率^[44]^。PCI的放射野包括全脑、脑干下到枕骨大孔或第一/第二颈椎基底部。

近期，两项前瞻研究^[45, 46]^显示总剂量25 Gy（分10次）或30 Gy（分15次）的PCI，对SCLC全身性治疗有明显益处，能明显提高患者总生存率，并适合于所有对化疗有效的LD和ED患者。

接受多重治疗时，PCI能加重认知功能损害，如：年龄相关性脑萎缩，先前存在的脑血管病、焦虑、抑郁及化疗毒副作用增加等^[47]^。因为PCI可增加疲劳、头痛、恶性/呕吐等神经毒性反应，故不建议同时应用全身化疗。

#### 2.4.2 靶向治疗

近年来，随着对肿瘤分子生物学的理解逐渐加深，靶向治疗正成为恶性肿瘤的重要研究方向。

组蛋白脱乙酰酶抑制剂（histone deacetylase inhibitor, HDACi）增加组蛋白和非组蛋白的乙酰化，从而在癌细胞生长和转移过程中调控基因和蛋白表达。在一项针对间皮瘤和多种肺癌细胞的研究^[48]^中，HDACi（LBH589）对SCLC显示了特殊效果，此外，LBH589与依托泊苷有协同抗癌作用。另一个HDACi——伏立诺他在临床前期和临床期研究中也显示了单独以及与拓扑替康协同治疗SCLC的作用^[49, 50]^。

在线粒体凋亡途径中，bcl-2蛋白家族具有关键调控作用，同时也与化疗耐受有关。约80%的SCLC过度表达bcl-2，表达水平与快速进展的恶性表型密切相关^[51]^。因此，bcl-2抑制剂在SCLC治疗中具有潜在应用价值，并对复发性SCLC展示了一定的疗效。

作为抑癌因子的早幼粒细胞白血病蛋白（promyelo-cytic leukemia protein, PML），在SCLC组织中低表达并与预后相关^[52]^；而PML转录后表达缺失可能源于蛋白激酶CK2的磷酸化以及泛素系统对它的异常降解所致^[53]^。因此蛋白激酶CK2有望成为SCLC的治疗靶点。

研究^[54]^发现聚合微粒体制剂NK012可以减轻顺铂治疗SCLC时产生的毒副作用。谷胱甘肽代谢和DNA修复基因的变异能够预测SCLC患者的生存，提示其具有成为潜在治疗靶点的可能^[55]^。

酪氨酸激酶抑制剂—吉非替尼、伊马替尼、厄洛替尼以及血管生成抑制剂—贝伐珠单抗、凡德他尼、索拉非尼、沙立度胺、Cediranib等现有靶向治疗药物中，除了贝伐珠单抗在联合EP方案治疗中显示了一定疗效外，其余药物均未显效。贝伐珠单抗联合化疗能提高ED患者的总生存率，并有较好的耐受性^[11, 56]^。

## 3 展望

1981到2008年，对比ED-SCLC一线全身化疗的Ⅲ期随机临床研究有52项。Oze等^[57]^对这些数据进行了线性回归和多因素分析。随着时间推移，入组患者生存时间没有延长，提示过去27年中ED-SCLC患者的生存状况没有改善。因此，需要开发新的治疗靶点、药物和综合治疗模式。

作为第一个被FDA批准的非鳞NSCLC维持治疗药物，培美曲赛是否适用于SCLC的维持治疗以及其它数十个问题正等待临床研究给出答案^[58]^。有理由相信，在不久的将来，更优化、有效的治疗方案会面世。同时也应看到，我国SCLC临床研究非常匮乏，因此，应加强这方面工作，尽早建立我国的SCLC临床指南。
